# How to Organise the Country

**Published:** 1919-12-20

**Authors:** 


### THE FINANCING OF THE VOLUNTARY HOSPITALS.

### How to Organise the Country.

During the last few weeks it has been made increasingly manifest that the difficulties attending the adequate financing of the voluntary hospitals throughout the country may probably be greater in 1920 and one or two subsequent years than ever before. To combat these difficulties the facts of the present position and their causes must be recognised and understood, first by those responsible for maintaining the voluntary hospitals in a state of the highest efficiency; and secondly by the people of all classes throughout each district who are dependent upon their local hospital or hospitals. From our constant intercourse and touch with all the interests concerned we are enabled to know with fair accuracy the circumstances of the position thus created.

One material fact in the present situation is that never before in the whole history of the voluntary system of hospitals have these institutions held a higher or stronger position in the minds and hearts of the men and women of all classes, who are in a position to give something, from 1s. upwards, in support of the institutions which have proved themselves throughout the country to be the best friends of the people. Further, this feeling of attraction for the voluntary hospitals, and of gratitude for and appreciation of the splendid work they do for the community, has been deepened and strengthened through the war, because it is a fact that our warriors and women workers who have gone to the Front, and proved such a splendid assistance in manifold ways as the war extended, have learnt from actual experience and their own observations, that the voluntary hospital, of all types of hospitals, is the very best place to make for when wounded or seriously ill, or in need of medical or surgical aid of any kind.

This practical universal awakening of interest in the voluntary hospitals, on personal and national grounds, on the part of armies of individual workers, who on demobilisation have returned or are returning to the districts where they habitually reside or work, has changed the hospital atmosphere and improved its quality. This is so because each hospital has in reality behind it thousands of men and women who would only be too glad to have an opportunity to work for this institution, and to do everything in their power to transfer the knowledge and experience they have to their neighbours and friends. Rejoicing at the Armistice, thankful for the return of their dear ones, and keenly expectant of the completion of Peace throughout the world, they would welcome an opportunity to celebrate its happy conclusion, and at the same time materially to aid and strengthen the institutions in their midst of which they are proud. Amongst all else, first and foremost, the voluntary hospital and all connected with it stand out. To them the population they serve owe so much.

In a word, the changed atmosphere, the new conditions, bring an army of awakened, educated, and keenly interested friends of the voluntary hospital into every centre of population throughout Great Britain. There is thus created a field, glowing in anticipation with the promise of harvest, of which the hospitals, owing to other war conditions, are in most urgent need, to enable the managers to adjust their finances by making good the losses due to the war, and adding sums adequate to bring every single one of these hospitals up to date, to extend its usefulness, and make all its equipment equal to the demands created by new conditions, advanced science, and the best interests of the patients who seek relief within its walls.

The serious outlook for the voluntary hospitals, if they had to depend solely on the old conditions and old supporters, who have stood by them with such commendable generosity in the past, cannot be denied. Many of these supporters have suffered grievously by the war, and have become unable, to their regret, to shoulder their old share of the burden of hospital maintenance, which they had borne with pride and pleasure when greater prosperity was theirs. In such circumstances either the powers of the hospitals, and the numbers that they could deal with must be diminished, or the devoted fraction of hospital supporters within a given area must be enlarged, by the recruiting of considerable numbers of the nine-tenths that remain, who up to the present time, have taken little or no interest in hospital work or hospital management.

The problem which presented itself was: How to Awaken and Secure the Interest of the Nine-Tenths of Indifferent Residents in a given centre who in reality constitute, from the financial point of view, fresh fields and pastures new. We have shown that a large section of the nine-tenths of fallow land have been changed by the war into cultivated fields, full of promise for the harvest, providing those interested in the hospitals awake and invite the cordial co-operation of returning warriors and noble women workers, who have recently returned to their homes, on demobilisation. Our investigations having confirmed the view we have here expressed, we immediately addressed ourselves to the Chairman of every London hospital and every hospital of 100 beds and upwards situated in Great Britain, inviting each to send a statement of (*a*) their needs at present and in the immediate future; and (*b*) their programme of development and extension, if any, during 1920, offering full publicity in the columns of The Hospital and every possible aid in our power to solve the problem of the hospital crisis, and make every one of the hospitals reasonably secure for the future. In our covering letter we made two points of importance, and drew attention to the way in which each hospital can help. We quote the paragraphs dealing with these matters.

### A DANGER TO BE MET AND OVERCOME.

In the latter months of 1919 irresponsible speakers here and there have started to cry "The Voluntary System is Doomed," and to suggest that the Voluntary Hospitals must be put upon the rates. Such utterances will not bear examination on the facts, and are contrary to the truth. They are proving mischievous, too, as the Treasurers of our voluntary hospitals know, for some of them have startling evidence of the danger which may arise, and result in serious loss to the revenue of the voluntary hospitals. Some of the wisest, most liberal, and constant supporters of our great hospitals resent the suggestion that the best system of hospitals in the world, which we possess, should be threatened with destruction, and a loss of efficiency, involving a set-back to progress and the uplifting and maintenance of the health of the people, should the voluntary hospitals be compelled to rely on State support for their upkeep and continuance.

### WANTED, THE GOVERNMENT'S ASSURANCE.

The truth is that all British statesmen recognise that the State has so much on its hands that any question of the nationalisation of hospitals, apart altogether from the merits or demerits of any such fate, must be postponed into the very dim future. A definite statement to this effect may be expected from the Government, or a member of the Government representing the Cabinet, seeing that a falling-off of revenue to the voluntary hospitals at the present time would seriously threaten the national finances and bring discredit upon the Prime Minister and his Government. No Government can hope for the support of the nation if it plays with working-class agitators who clamour for the establishment of Rate Hospitals and the casting of the Voluntary Hospitals on the Rates.

### HOW EACH HOSPITAL CAN HELP.

**"The Hospital" continues to stand, as it has ever stood, as the champion of the voluntary hospital system which cannot and will not be allowed to die. It confidently awaits the account you may send, in response to this letter, to show what your centre is doing, or about to do, at this fateful time.**

**The response of the Chairmen has been wonderful, as the reader may judge for himself by perusing the replies received, which are given on pages 269 to 276.**

It remains to organise locally the inhabitants dependent on a particular hospital or hospitals, and *The Times* has lent its powerful influence to the aid of the voluntary hospitals by giving prominence, in its issue of the 17th inst., to the letter on Hospital Finance which we reprint in full on this page. Hospital managers throughout Great Britain have at present the opportunity to win a great victory for the voluntary hospitals, and to secure adequate means to provide the financial strength which they lack. All that is wanted is courage, energy, conviction, and the co-operation with renewed strength of those who have made good in the war and are now home. We are in a position to add to these aids to triumphant success, a guarantee to hospital managers everywhere, who may choose to avail themselves of it, of the Administrative Aid which secured the adequate organisation of the King's Hospital Fund, the wonderful yield of which is strikingly demonstrated by the publication of the proceedings at the twenty-second annual meeting of that Fund, held at St. James's Palace, on December 16, see page 261.

